# Unpacking the Complexity of Epithelial Plasticity: From Master Regulator Transcription Factors to Non-Coding RNAs

**DOI:** 10.3390/cancers15123152

**Published:** 2023-06-11

**Authors:** Charlene Waryah, Eric Alves, Roberta Mazzieri, Riccardo Dolcetti, Erik W. Thompson, Andrew Redfern, Pilar Blancafort

**Affiliations:** 1 Cancer Epigenetics Group, Harry Perkins Institute of Medical Research, Perth, WA 6009, Australia; 2 School of Human Sciences, University of Western Australia, Perth, WA 6009, Australia; 3 Peter MacCallum Cancer Centre, Melbourne, VIC 3000, Australia; 4 Sir Peter MacCallum Department of Oncology, The University of Melbourne, Melbourne, VIC 3010, Australia; 5 Department of Microbiology and Immunology, The University of Melbourne, Melbourne, VIC 3010, Australia; 6 School of Biomedical Sciences, Faculty of Health, Queensland University of Technology, Brisbane, QLD 4059, Australia; 7 Translational Research Institute, Brisbane, QLD 4102, Australia; 8 School of Medicine, University of Western Australia, Perth, WA 6009, Australia

**Keywords:** cellular plasticity, epithelial plasticity, epigenetics, epithelial to mesenchymal transition, transcription factors, non-coding RNAs, immune evasion

## Abstract

**Simple Summary:**

Epithelial-to-mesenchymal transition (EMT) is a complex program by which epithelial cells lose epithelial characteristics whilst acquiring mesenchymal features. EMT was coined in the 1980s and initially thought to involve a binary switch between epithelial and mesenchymal states. However, mounting work suggests that EMT involves intermediate states or hybrid epithelial/mesenchymal (E/M) phenotypes. In the context of many cancers, such as breast cancers, these hybrid states retain characteristics of both epithelial and mesenchymal cells, and have been linked to poor survival, metastasis, and resistance to treatment. In this Review, the authors examine the complex layers of molecular interactions governing EMT in cancer. The underlying drivers of these states, namely EMT-transcription factors, epigenetic regulators, and non-coding RNAs, as well as the influence of EMT on the immune response, are discussed, and in doing so, this Review outlines valuable mechanistic insights for the reversion of EMT and potential avenues for therapeutic intervention.

**Abstract:**

Cellular plasticity in cancer enables adaptation to selective pressures and stress imposed by the tumor microenvironment. This plasticity facilitates the remodeling of cancer cell phenotype and function (such as tumor stemness, metastasis, chemo/radio resistance), and the reprogramming of the surrounding tumor microenvironment to enable immune evasion. Epithelial plasticity is one form of cellular plasticity, which is intrinsically linked with epithelial–mesenchymal transition (EMT). Traditionally, EMT has been regarded as a binary state. Yet, increasing evidence suggests that EMT involves a spectrum of quasi-epithelial and quasi-mesenchymal phenotypes governed by complex interactions between cellular metabolism, transcriptome regulation, and epigenetic mechanisms. Herein, we review the complex cross-talk between the different layers of epithelial plasticity in cancer, encompassing the core layer of transcription factors, their interacting epigenetic modifiers and non-coding RNAs, and the manipulation of cancer immunogenicity in transitioning between epithelial and mesenchymal states. In examining these factors, we provide insights into promising therapeutic avenues and potential anti-cancer targets.

## 1. Introduction

Epithelial cells have defined structural features which include the polarized distribution of the plasma membrane components and uniform cell–cell junctions, giving rise to a wide array of cellular states. Intercellular adhesions provide epithelial cells with intrinsic and structural “rigidity”. On the other hand, mesenchymal cells lack such uniformity with decreased structural integrity and adhesions. Unlike their epithelial counterparts, mesenchymal cells exhibit elongated, irregular morphologies, and exhibit an ability to migrate and invade through the extracellular matrix.

The phenomenon by which epithelial cells undergo complex phenotypic changes and acquire mesenchymal features is referred as epithelial-to-mesenchymal transition (EMT). This process was first coined in 1982 by Elizabeth Dexter “Betty” Hay and her team, who described the ability of epithelial cells to switch “off” epithelial genes while acquiring mesenchymal characteristics [1]. This pioneering work observed that cultured chick embryo epithelial cells had a remarkable ability to move and migrate through the collagen matrix in which they were suspended. These migratory cells displayed mesenchymal properties and were described as “transformed”. Further investigation detailed that reverse changes could occur in these migratory cells where they would anchor and regain epithelial-like characteristics.

EMT and its reverse process, mesenchymal-to-epithelial transition (MET), is instrumental in developmental programs, including embryonic association with implantation and embryonic development (type I EMT) and wound healing [2]. However, abnormal activation of EMT programs is induced by conditions such as fibrosis (type II EMT) and cancer (type III EMT) [2,3]. The different types of EMT have been extensively reviewed by Kalluri and Weinberg, 2009 [2]. For decades, EMT was viewed to comprise two distinct cell types—epithelial and mesenchymal—with a binary switch between the two states. However, in 2020, a consensus among EMT researchers established that this biological phenomenon encompasses a spectrum of dynamic intermediate states of bidirectional EMT to MET giving rise to epithelial–mesenchymal plasticity (EMP) [4].

There are several regulatory processes or layers of complexity that modulate EMP. At the core of EMP lies pro-mesenchymal transcription factors (EMT-TFs). EMT-TFs specifically regulate their target genes via consensus DNA-binding domains, which vary across the main families of EMT-TFs. Furthermore, their N- and C-terminal effector domains promote the association with particular partners, and the recruitment of a constellation of different epigenetic modifiers, modulating gene activation and/or repression. Added layers of regulatory mechanisms include non-coding RNAs, such as micro RNAs (miRNAs), many of which are involved in regulatory feedback loops with the EMT-TFs themselves, thus greatly amplifying the scope of targets implicated in EMP. Beyond these molecular processes intrinsic to cancer cells, there are also cross-talk mechanisms established between the cancer cells, the surrounding tumor microenvironment, and the infiltrating immune response, which can further modulate EMP.

**Figure 1:**
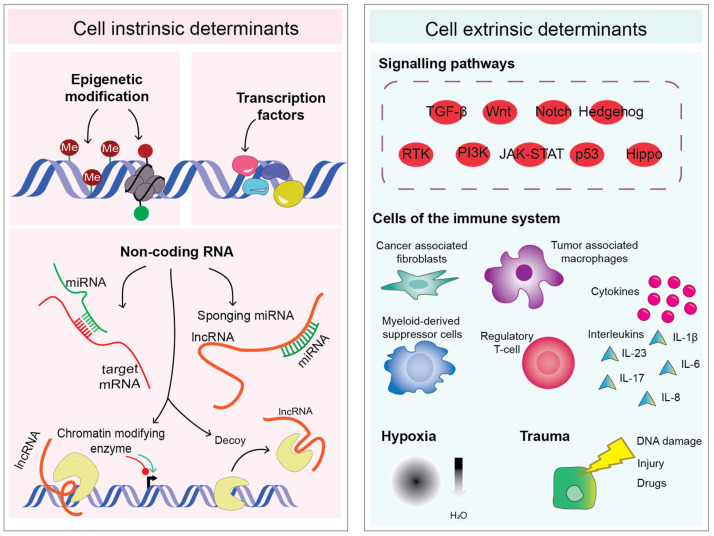
Schematic diagram displaying the complexity of cancer cell plasticity and regulation, which can be divided into two major categories of triggering factors: cell intrinsic and cell extrinsic determinants. Me: DNA methylation, miRNA: microRNA, lncRNA: long non-coding RNA, TGF-β: transforming growth factor-β, Wnt: Wnt signaling, RTK: receptor tyrosine kinases, PI3K: phosphoinositide 3-kinase, JAK-STAT: Janus kinase-signal transducer and activator of transcription, IL: interleukin.

Carcinoma cells in the form of transformed cell lines and solid tumors are notoriously heterogeneous, and can be positioned in particular cell states along a continuum of a bi-directional plasticity axis. Importantly, this axis includes a range of quasi-epithelial and quasi-mesenchymal phenotypes existing in equilibrium [5]. Loss of epithelial E-cadherin and gain of mesenchymal N-Cadherin, Fibronectin, and Vimentin have been long considered hallmarks of EMT [6]. Importantly, during tumorigenesis and cancer progression, tumor cells rarely attain a complete mesenchymal phenotype [7]. Instead, cancer cells undergo commonly partial EMT (pEMT) resulting in E/M hybrid states where the same cell co-expresses epithelial and mesenchymal markers [7,8,9]. The positioning of cancer cells along the EMP axis is defined by an interplay of several EMT inducers and regulators, outlined in Figure 1. 

Much of EMT research has focused on the upregulation of “core” EMT-TFs in cancer cells, such as zinc-finger E-box-binding (ZEB) 1 and 2, snail family transcriptional repressor 1 (SNAI1, SNAIL), snail family transcriptional repressor 2 (SNAI2, SLUG), and twist-related protein (TWIST) 1 and 2 [10]. These EMT regulators cooperate to suppress pro-epithelial genes, particularly those involved in tight junction and adhesion. In addition, several non-coding RNAs, such as pro-epithelial miRNAs [11], are involved in regulatory feedback loops with EMT-TFs, and are key for specifying the different cell states along the EMP axis. One prime example is the double negative feedback loop between members of the microRNA-200 family (miR-200a, miR-200b, miR-200c, miR-429, and miR-141) with the ZEB1/ZEB2 EMT-TFs [12,13]. 

In addition to the “intrinsic” regulation of EMT-TFs in cancer cells, various extracellular signals and intracellular pathways associated with an aggressive tumor microenvironment (TME) modulate EMT either directly or indirectly, including the cytokine transforming growth factor-β (TGFβ), tumor necrosis factor-α (TNFα), Notch, JAK/STAT, Wnt/β-Catenin, and PI3K/Akt/mTOR [14,15,16,17,18]. These pathways, together with other regulatory cues, can activate EMT-TFs, often in combination with suppression of pro-epithelial miRNA expression, thereby facilitating cancer cell survival and metastasis [19,20].

Not surprisingly, hybrid E/M states, characterized by co-expression of epithelial and mesenchymal markers, have been associated with poor prognosis cancers with the ability to metastasize and resist treatment [7,21,22]. Gene expression profiling has identified several EMT-related gene signatures in numerous cancer types, which facilitate the scoring and positioning of these hybrid cancers along an EM spectrum [23,24,25]. A crucial property of hybrid cancer cell populations is the ability to escape the killing by cytotoxic immune cells. Cancer cells in various states of EMT secrete an array of cytokines, chemokines, and growth factors, which influence the differentiation, homing, and activity of various immune cell populations [26,27,28,29,30]. Below, we discuss the intricate cross-talk between layers of epithelial plasticity in cancer, including the core layer of transcription factors, their interaction with epigenetic modifiers and non-coding RNAs, and the manipulation of tumor immunogenicity during the transition between epithelial and mesenchymal states.

## 2. Epigenetics and the Transcription Factor Network

**Figure 2:**
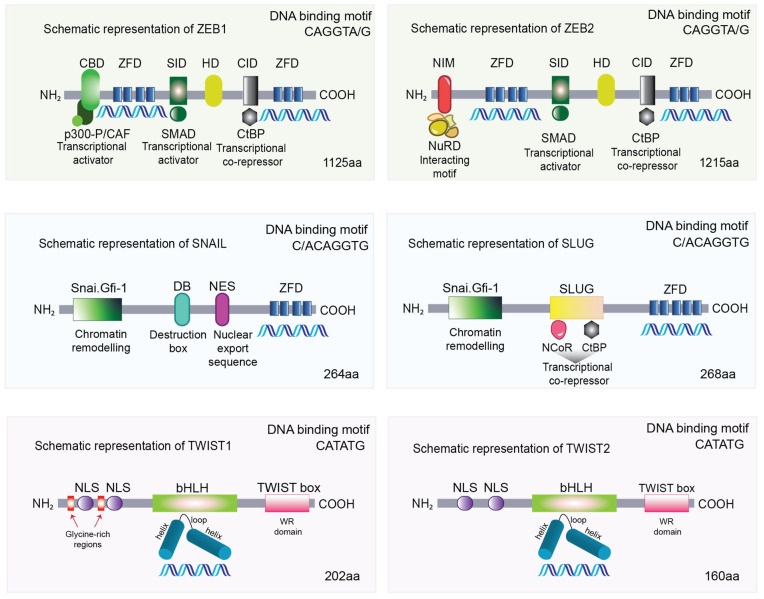
Human ZEBs, SNAIL, SLUG, and TWISTs protein structure and interacting domains. NH_2_: N-terminus, COOH: C-terminus, CBD: CAF/P300 binding domain, ZFD: zinc finger domain, SID: Smad interaction domain, HD: Homeodomain, CID: CtBP interaction domain, NIM: NuRD interacting motif, NuRD: nucleosome remodeling and deacetylase complex, Snai.Gfi-1: SNAG domain, DB: destruction box, NES: nuclear export sequence, NCor: nuclear receptor co-repressor, NLS: nuclear localization signals, bHLH: basic helix-loop-helix, aa: amino acids.

Although epigenetic modifications in cancer cells have been implicated in EMT, the extensive epigenetic reprogramming network driving plasticity is not fully understood. By definition, TFs are proteins that respond to stimuli from the extracellular environment and bind to specific DNA sequences with consequent regulation of gene expression. Therefore, TFs can stimulate or inhibit target gene expression during EMT induction and cancer progression [31]. Functionally, TFs involved in regulating epithelial plasticity are collectively referred to as EMT-TFs [32,33]. EMT-TFs bind specifically to their target genes through their different DNA-binding domains (Figure 2). Altered EMT-TF expression is frequently observed in cancers and studies have highlighted their contribution to cancer biology through various mechanisms. In addition to their DNA binding domains, EMT-TFs also vary in structure, as manifested by their different effector domains interacting with various epigenetic modifiers. These “epimodifiers” induce epigenetic modifications and chromatin remodeling in their target DNAs, leading to either gene activation or gene repression, as discussed in detail below (Figure 2).

### 2.1. SNAIL and SLUG

The first EMT-TFs molecularly characterized were SNAIL and SLUG [34,35]. Both SNAIL and SLUG contain tandem zinc-finger motifs on the *C*-terminus and a Snai/Gfi-1 (SNAG) repressor domain on the *N*-terminus (Figure 2). While differences exist with SNAIL containing four C_2_H_2_ zinc-finger motifs (cysteine/histidine, coordinating a zinc ion) and SLUG containing five, both share a high degree of homology and function as DNA-binding motifs targeting consensus E2-box type elements (C/ACAGGTG) [36]. Their SNAG domain is essential for nuclear localization and, on DNA binding, functions as a molecular hook to recruit co-repressors and epigenetic remodeling complexes, thereby exerting their role as transcriptional repressors. SNAG recruits histone lysine specific demethylase 1 (LSD1), which removes mono- and di-methylation at lysine 4 on histone H3 (H3K4me, H3K4me2) [37]. LSD1 is frequently associated with the CoREST ternary complex, which additionally associates with histone deacetylase HDAC1/2. Furthermore, LSD1 may have a dual role since, in addition to repression, it has been shown to alter H3 lysine 9 state by removing mono- and di-methylation, resulting in transcriptional activation [38].

The SNAG domain of SNAIL has also been shown to interact with HDAC1, HDAC2, and the co-repressor SIN3 transcription regulator homolog A (mSin3A) [39]. Further, in human breast cancer, SNAIL has been shown to interact with histone methyltransferase G9a inducing H3K9me2, and further recruit both G9a and DMNTs to the *CDH1* promoter to induce DNA methylation and gene silencing [40].

In breast cancer, SLUG forms a complex with LSD1 and protein arginine methyltransferase 5 (PRMT5), which facilitates cancer invasion [41]. In addition, SNAIL can induce the repressive H3K27me3 mark via the recruitment of PRC2 together with the co-regulators LIM protein AJUBA and PRMT5 [42]. 

Differences between SNAIL and SLUG lie in the inclusion of a SLUG domain, an additional unique 28-amino acid sequence only present in the latter. Functional studies have found that SLUG interacts with the co-repressors NCoR and CtBP1, and deletion of the SLUG domain fully abolishes interaction with CtBP1 [43]. While the exact mechanism of the SLUG domain binding to epimodifiers is yet to be uncovered, this additional sequence renders the SLUG protein susceptible to post-translational modifications affecting proteolytic function and/or cellular localization [44]. 

The most notable target of both SNAIL and SLUG is the promoter of the *CDH1* gene, which encodes the quintessentially epithelial protein, E-cadherin. Interaction between SNAIL and SLUG with epimodifiers through their SNAG domain is essential for repression of E-cadherin (*CDH1*). SNAIL and SLUG also bind to E-boxes in other key target epithelial genes in human breast cancer including *claudin-1* [45], *Muc1* [46,47], *Vitamin D receptor* [48], *integrins* [49], *cytokeratins*, and *occludins* [50,51]. 

In addition to epithelial proteins, SNAIL and SLUG are involved in the epigenetic repression of tumor suppressor genes involved in EMT. For example, SNAIL and SLUG have been shown to bind to E boxes (enhancer box) in the *PTEN* promoter, thereby negatively regulating *PTEN* expression [52,53,54]. Repression of *PTEN* contributes to the development of tumorigenesis, and resistance to PI3Kα inhibitors and targeted therapies, such as trastuzumab or doxorubicin [52,55,56,57]. Furthermore, PTEN exerts its tumor suppressive functions by inhibiting the PI3K-AKT pathway, which also controls EMT [52]. In contrast, SLUG is a negative regulator of *BRCA2* in human breast cancer via recruitment of CtBP1 and HDAC1 at E boxes in the *BRCA2* promoter [58]. 

### 2.2. TWIST1 and TWIST2

The basic helix-loop-helix (bHLH) family of proteins include a range of TFs containing a conserved domain characterized by two α-helices connected by a short inter-helical loop (Figure 2) [59]. These proteins are further classified into three subfamilies: classes A, B, and C. Class A proteins are ubiquitously expressed, whereas class B proteins have tissue specificity. In many instances, class B proteins form dimers with proteins of class A through interactions mediated by α-helices. This dimerization permits the binding to specific hexanucleotide E-box sequences (CATATG) leading to transcriptional regulation of target genes [60]. Twist-related proteins 1 and 2 (TWIST1 and TWIST2) fall into class B as they form dimers with class A proteins, particularly E proteins such as E12 and E47, which are critical regulators of B cell development [61]. Additionally, class B members form dimers with proteins belonging to the same class. Within the bHLH domain of class B TWIST lies a Thr-Gln-Ser (TQS) motif vital for promoting metastasis [62]. Upon phosphorylation, this motif modulates binding affinities with partner proteins specifying cell fate [63]. 

The TWIST1 and TWIST2 TFs have 100% structural similarity in the C-terminus Twist box, and 95% similarity in the bHLH region, while only 54% similarity in the N-terminus. The N-terminus contains two nuclear localization signals (NLS), mediating effective translocation to the nucleus required for DNA binding. The traditional view of mammalian TWIST function is that of inhibiting differentiation of mesenchymal cell lineages such as inhibition of myogenesis and osteogenesis. TWIST1 and TWIST2 display a bifunctional role as activators or repressors depending on the binding partner choice, chromatin accessibility and specific cell type. Overall, target promoters for TWIST1 and TWIST2 have multiple E-boxes suggesting combinations of several dimers could influence binding, thereby controlling transcriptional outcome. 

The C-terminus 20 residue Twist box (also known as WR domain) is critical for TWIST transcriptional activity [64]. The TWIST1 N-terminal region contains an additional two glycine rich tracks rendering TWIST1 protein larger than TWIST2. Physical interaction between TWIST1 and p300, cAMP-response element binding protein (CREB), CREB-binding protein (CBP), and p300/CBP-associated factor (PCAF) is mediated by this N-terminal glycine-rich tracks that block histone acetyltransferase (HAT) activity [65]. TWIST2, lacking these additional glycine-rich tracks, does not have the ability to block HAT. Yet, both TWIST1 and TWIST2 have the ability to recruit HDACs, with TWIST1 previously shown to interact with several components of the Mi2/nucleosome remodeling and deacetylase (Mi2/NuRD) complex and Rb-associated protein 46 [66,67,68]. Of note, it has been demonstrated that TWIST1 in complex with E12 recruits Mi2/NuRD/MTA2 complex and directly interacts with MTA2 to repress *CDH1* expression [66,69].

Both TWIST1 and TWIST2 also interact with other TFs including SMAD4, MyoD, MEF2, RUNX1/1, CEBP-α, and NF-ĸB [68,70,71,72,73,74,75]. Furthermore, TWIST1/2 proteins have the potential to interfere with the transcriptional activity of MyoB and MEF2 TFs, thereby inhibiting myogenic differentiation.

### 2.3. ZEB1 and ZEB2

The zinc-finger E homeobox-binding (ZEB) family of TFs contains two members, ZEB1 and ZEB2, which play a central role in cancer cell plasticity. ZEB proteins not only control global (genome-wide) reprogramming of plasticity [32,76,77], but also specific pathways including Hippo [78], Notch [79], and NF-κB [80]. 

Structurally, the ZEB proteins are highly homologous in the two C_2_H_2_ zinc-finger motifs located at the N- and C- termini (Figure 2). Both ZEB members bind to the paired E2-box type elements (CAGGTA/G) [81]. Along with the centrally located homeodomain, the zinc fingers mediate ZEB1 and ZEB2 binding at various target genes. Further, the interaction with repressive epimodifiers occurs via protein–protein binding domains including the SMAD binding domain and the CtBP interaction domain, both near the N-terminus of the ZEB TFs (Figure 2). Interestingly, ZEB1 contains binding domains for both p300 and P/CAF (close to the C-terminus) as well as an activation domain (N-terminus) rendering the protein a potential transcriptional activator. Interestingly, binding of ZEB1 with P/CAF acetylates lysine resides close to the N-terminal CtBP interaction domain, switching it from a transcriptional repressor to an activator, a feature which is not observed with the ZEB2 TFs. 

Through its CtBP interaction domain, ZEB TFs are capable of interacting with the CtBP transcriptional co-repressor [82], which recruits histone deacetylases HDAC1/2. Depending on the chromatin context, in addition to HDAC1/2, CtBP-associated proteins also include histone methyltransferases G9a and EuHMT, chromodomain-containing proteins HPC2 and CDYL and CoREST [83]. While CtBP plays a key role in the ZEB1/2-mediated repression of *CDH1*, ZEB1 specifically acts as a transcriptional repressor via its interaction with BRG1 (BRM/SWI2 related gene 1), a component of the SWItch/sucrose non-fermentable (mSWI/SNF) chromatin remodeling complex. This complex has the capacity to bind to the minor grooves in DNA and utilize ATP to perturb histone–DNA interactions affecting the target chromatin structure [84]. 

SMADs are a group of intracellular proteins which are the main transducers of TGFβ signaling receptors, and are able to regulate gene transcription. The differential recruitment by ZEBs by SMAD proteins is evidenced by their powerful role as regulators of the TGFβ and BMP signaling pathways, which are involved in several cellular processes including cancer progression [85,86]. Members of the secretory TGFβ cytokine family, including TGFβ and BMP, stimulate the induction of SMAD proteins leading to nuclear translocation. Both ZEB1 and ZEB2 proteins bind to receptor regulated SMADs through their SMAD binding domain; however, ZEB1/2 have been shown to have antagonistic effects [87]. Once in complex, ZEB1 synergizes with SMAD-mediated transcriptional activation of downstream targets while ZEB2 represses it. 

The ZEBs are best known as key drivers of EMT by repressing *CDH1* and the pro-epithelial miR-200 family members. Downstream targets of ZEB1 and ZEB2 have been well studied in the context of breast cancer and include key genes involved in epithelial plasticity and cellular polarity genes [88,89,90]. By targeting promoters of pro-epithelial genes, ZEBs are dominant regulators of EMT and tumor progression. High ZEB expression is associated with poor prognosis, metastasis, and resistance to chemotherapy in a variety of cancer types [91]. Furthermore, there is increasing evidence of ZEB-mediated regulation of inflammatory responses, thereby supporting the TME and potentiating tumor growth [92]. 

## 3. Transcription Factor Cooperative Regulation

**Figure 3:**
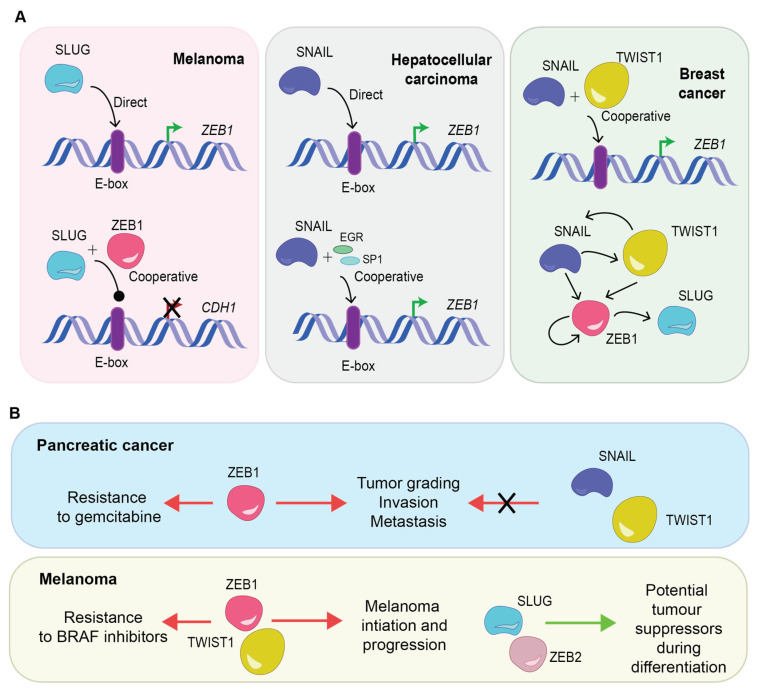
(**A**) Schematic diagram demonstrating the interaction between EMT-TFs in different cancers with direct, indirect, and cooperative interactions observed. (**B**) Examples of the differential roles EMT-TFs can play in resistance with oncogenic functions, but may have the potential to act as tumor suppressors. E-box: enhancer box, EGR: transcription factor early growth response factor 1, SP1: transcription factor specificity protein 1.

The EMT-TFs regulate important genes controlling cancer initiation, development, therapy resistance, and metastasis [93]. They have also been shown to functionally cooperate and regulate one another (Figure 3A). For example, in melanoma, ChIP assays identified SLUG as a direct activator of ZEB1 with the ability to bind to E-boxes within the ZEB1 promoter [94]. Whereas in hepatocellular carcinoma, SNAIL can both directly and indirectly activate ZEB1 transcription [95]. Similarly, in mouse breast epithelial cells treated with TGF-β to induce EMT, SNAIL, and TWIST cooperate in inducing ZEB1 transcription [96]. In the same study, SNAIL was able to increase both TWIST1 protein expression and stability. 

Although EMT-TFs broadly converge to induce EMT programs, recent data supports the notion that each EMT-TF is involved in specialized, non-redundant and/or tissue-specific roles, orchestrating different functions in cancer pathobiology [97,98,99]. This intricate involvement, which remains elusive in many cancer types, fuels carcinoma cells with the plasticity to differentially activate many transcriptional programs and epigenetic landscapes. This is particularly important in the context of resistance and adaptation to particular insults, such as metabolic stress, DNA damage caused by chemotherapies and radiation, as well as other mechanisms of therapy-induced resistance [4,97,100].

It has been proposed that the capacity of carcinoma cells to exist in hybrid E/M phenotypes is controlled by the differing molecular roles and thus the differential expression of each EMT-TF [97,98]. For example, to drive hybrid E/M tumors to a fully mesenchymal phenotype in breast carcinoma cells requires ZEB1 expression. Not surprisingly, in breast tumors, ZEB1 expression levels are highly elevated in mesenchymal cell states [98]. In contrast, the highly tumorigenic hybrid E/M cell state is driven by SNAIL with more than five-fold protein increase in hybrid E/M states compared to mesenchymal cells. Whereas TWIST levels were elevated in both hybrid and mesenchymal populations as compared to epithelial cell states [98].

Importantly, there are specialized roles EMT-TFs play in resistance to a range of chemotherapies. In ovarian cancer, upregulation of SNAIL and SLUG has been directly correlated with resistance to cisplatin [101], paclitaxel [102], and radiation [102], as well as gefitinib resistance in lung cancer cell lines [103]. ZEB1 induces resistance to epirubicin in breast cancer [104], oxaliplatin in esophageal cancer [105], docetaxel in prostate cancer [106], and gemcitabine in pancreatic cancer [107]. In addition to resistance to particular agents, EMT can be induced after various treatments in a bid to adapt to cellular stress induced by treatments including chemo-, radio- and immune-based therapies [108].

While individual signals that trigger EMT have been identified, our understanding of the impact of the differential EMT-TF expression in the different cancer phenotypes remains elusive. However, some nuances have been characterized, as outlined in Figure 3B. In pancreatic cancer, depletion of ZEB1 affected tumor grading, invasion, and, importantly, metastasis in contrast with SNAIL and TWIST1 [32]. In breast cancer, however, SNAIL may also trigger metastasis [109]. During melanocytic differentiation, both SLUG and ZEB2 act as tumor suppressor proteins whereas ZEB1 and TWIST1 are oncogenic proteins driving melanoma initiation and progression [110]. This demonstrates the enormous flexibility of the biological effects of EMT-TFs in inducing metastasis and resistance to particular agents, depending on the cancer type. This flexibility could also be explained by tissue specific expression of EMT-TF partner proteins and the vast array of epigenetic modifiers each TF can recruit.

## 4. EMT-TFs Are Induced by Proinflammatory Mediators

In the same way that cellular plasticity provides cancer cells with the ability to adapt to the TME, this characteristic additionally confers the capacity to escape immune detection and elimination. EMT-TFs are central to the deployment of immune evasion mechanisms in cancer cells, which can be induced in the presence of chronic inflammation—a hallmark of cancer. In this context, further to the aforementioned TGF-β interaction [111,112], activation of EMT-TFs have been shown to be closely associated with other key pro-inflammatory mediators, including IFNγ [113,114], IL-1β [115,116], IL-6 [117,118,119], IL-8 [119,120], IL-1 [121], IL-23 [122], CCL2 [123,124], CCL5 [125,126], and CCL18 [127]. Together, these molecules promote chronic inflammation in the TME, and ultimately, foster the acquisition of EMT-like features in cancer cells. The origin of these inflammatory mediators varies within the tumor and may arise from various cell types, including polarized “M2-like” (Arg1^high^/CD206^high^/IL-10^high^) pro-tumoral macrophages (tumor-associated macrophages; TAMs) [128], myeloid-derived suppressor cells (MDSCs) [129], CD4^+^Foxp3^+^ regulatory T cells (Tregs) [130], and/or the tumor cells themselves [112]. Importantly, the persistent inflammatory environment within the tumor produced by these cells attracts additional immunosuppressive cells into the TME, promoting continued expression of EMT-TFs, and results in a detrimental positive feedback loop that drives tumor progression and inhibits anti-tumor immunity. For example, work by Su and colleagues [131], has shown that mesenchymal-like breast cancer cell lines have the capacity to polarize macrophages to an “M2-like” phenotype via their secretion of granulocyte-macrophage colony-stimulating factor (GM-CSF). These TAMs, in turn, produce elevated levels of CCL18, which induces EMT in breast cancer cells via the phosphatidylinositol 3-kinase (PI3K)/Akt/glycogen synthase kinase 3β (GSK3β)/SNAIL signaling pathway. This then initiates a positive feedback loop between GM-CSF from breast cancer cells and CCL18 from TAMs that was shown in their humanized mouse model to drive tumor metastasis in vivo. Furthermore, other studies have shown that sustained production of GM-CSF by tumor cells also promotes the induction or recruitment of MDSCs, which inhibit CD8^+^ T cell function and detrimentally impact the overall anti-tumor immune response [132,133]. These studies highlight that interactions between tumor cells and other cells in the TME are highly complex and have the capacity to significantly impact the anti-tumor immune response.

## 5. EMT-TFs Facilitate Immune Evasion

In recent years, major advancements have been made in the development and application of cancer immunotherapies, including immune checkpoint inhibitors (ICIs) and adoptive cell therapy (ACT). However, despite these breakthroughs, low response rates and therapeutic resistance remain major obstacles to achieving clinical benefit across patients, particularly in solid cancer types. The poor response and acquired resistance seen in some patients is, in part, due to EMT-driven immune evasion and tumor-related immunosuppression. Although current cancer immunotherapies have the capacity to target major immune checkpoints such as PD-1, PD-L1, or CTLA-4 to galvanize the anti-cancer T cell response [134,135,136], numerous other immune regulatory checkpoints are also upregulated on the surface of EMT cancers, including CD73 [137], CD155 [138], PD-L2 [139], and HVEM [140], which are not addressed by current ICI therapies. Activation of these alternative immune checkpoints hinders effective anti-tumor immune responses.

Moreover, EMT-TFs have been shown to be associated with reduced expression of major histocompatibility complex (MHC) Class I [141,142], which plays a pivotal role in the presentation of tumor antigens on the tumor cell surface for recognition by CD8^+^ T cells and activation of cytotoxic T cell responses [143]. In addition to reduced MHC Class I expression, Tripathi and colleagues [144] showed that EMT in lung cancer cell lines results in reduced expression of various immunoproteasome components (IRF1, STAT1, PSMB8, PSMB9, and PSMB10), which are required for efficient antigen processing and presentation by MHC Class I. This depletion of the immunoproteasome was found to reduce the repertoire of MHC Class I-bound peptides and impede cancer cell recognition and killing by CD8^+^ T cells. Interestingly, immunoproteasome deficiency and lack of MHC Class I was restored in this study by treatment with the inhibitor of DNA methylation 5-aza-2′-deoxycytidine, suggesting an epigenetic mechanism of repression in EMT to facilitate immune evasion. Alternatively, other studies have shown that direct knockout of EMT-TFs can abrogate tumor immunosuppression, improve local infiltration of CD8^+^ T cells, and induce systemic anti-tumor immune responses [112].

Aside from MHC Class I, EMT-TFs have previously been shown to activate the inhibitory immune receptors killer Ig-like receptors (KIR) KIR3DL1, KIR2DL1, KIR2DL3, and KIR2DL4 [145], and repress activating immune-receptor natural killer group member D (NKG2D) ligands UL-16 binding protein (ULBP) 1 [146] and ULBP2 [147] on the tumor cell surface. Interestingly, Lopez-Soto and colleagues [147] showed that induction of EMT in colorectal cancer cells via SNAIL overexpression induced upregulation of the NKG2D ligands MICA/B and ULBP2, suggesting an avenue for NKG2D-mediated natural killer (NK) cell immunotherapy [148]. However, follow-up experiments identified that levels of soluble MICA were also elevated in the SNAIL-activated tumors, which act as a decoy to limit anti-cancer surveillance by NK cells. This highlights the potential for different evasion mechanisms to be simultaneously involved in the context of EMT. 

## 6. Non-Coding RNAs Add Another Layer of Intricacy

Until recently, the role of non-coding RNAs (ncRNAs) in EMT was limited. With the realization of their involvement in numerous diseases, such as cancer, there has been a plethora of interest in their role and functions in epithelial plasticity. Non-coding RNAs are functional RNA moieties that do not require translation into protein for their action [149].

Although there are several categories of ncRNAs, they are loosely divided into two main groups based on their length. One of the most studied groups of ncRNAs are microRNAs (miRNAs), which are small, single-stranded non-coding RNAs approximately 18–22 nucleotides in length. MicroRNAs play a crucial regulatory role in the behavior of every biological process as they contain a “seed sequence” complementary to the 3′UTR (untranslated region) of target mRNA, which in most cases facilitates binding and degradation. Under certain conditions, miRNAs have also been shown to induce gene expression [150,151,152].

There are over 2600 known mature miRNAs (miRBase v2.2 [153]) which are involved in the regulation of >60% of human protein coding genes. In cancer, they have been shown to act as either oncogenes or tumor suppressors. Numerous miRNA signatures have been described for various stages of cancer initiation and progression. Therefore, in-depth research has been directed to understanding the role of miRNAs in cancer progression and metastasis. The role of miRNAs in EMT-TF regulation is well established and many of them participate in feedback loops with EMT-TFs [11,19,154]. Therefore, miRNAs’ regulatory interaction with TFs are crucial contributors of cancer cell plasticity. 

In particular, the negative feedback loop of members of the miR-200 family with the ZEBs has been extensively investigated [13,155]. Members from the miR-200 and miR-205 families can repress expression of ZEB1 and ZEB2 by binding to 3′ untranslated regions of mRNA, thus preventing translation [12,13]. There is also a feedback loop between miR-34 and SNAIL. In the SNAIL 3′UTR lies a conserved seed sequence facilitating binding of *miR-34a/b/c*. Ectopic miR-34a results in direct downregulation of *SNAIL* mRNA and protein resulting in the induction of MET [156]. Other well-established interactions include miR-137/SNAIL, let-7/SNAIL, miR-218/SLUG, miR-218/ZEB2, miR-203/SNAIL, and miR-203/SLUG (extensively reviewed in [19,154,157,158]). 

Considering their role in both tumor pathogenesis and EMT modulation, miRNAs are emerging as epigenetic players in cancer immune evasion. Multiple miRNA (>50) regulate the expression of PD-L1, such as miR-142 in pancreatic cancer [159], miR-138 in colorectal cancer [160], and miR-570 in hepatocellular carcinoma [161]. Similarly, in lung cancer, regulation of PD-L1 expression has been linked to the miR-200/ZEB1 axis [134]. Additionally, indirect epigenetic regulation of PD-L1 expression by miRNAs occurs through regulation of signaling pathways, including activation of STATs, PI3K/Akt, and suppression of PTEN [162]. A direct inhibitor of SNAIL and EMT in breast and ovarian cancers, miR-34a inhibits the expression of PD-L1 in acute myeloid leukemia [163].

Unlike their smaller ncRNA counterparts, long non-coding RNAs (lncRNA) are underexplored and remain an untapped resource in cancer therapeutics. With advancement in transcriptomic profiling, there is mounting evidence of lncRNA contribution in modulating EMT-TFs, thereby affecting regulation of cancer cell plasticity [164,165,166,167,168,169]. LncRNAs vary in length, ranging from 200 nucleotides up to ~100 kilobases and do not translate into proteins. In prostate cancer, antisense lncRNA ZEB1-AS1, which is co-expressed with ZEB1, epigenetically activates ZEB1 by recruiting the histone methyltransferase MLL1 [170]. Interestingly, lncRNA ZEB2-AS1 contains a complementary sequence to a splice site located in the 5′UTR ZEB2 intron. This particular region in the intron contains an internal ribosome entry site. Upon ZEB2-AS1 binding, splicing of this region is disrupted, thereby improving ZEB2 translation efficiency and increasing overall ZEB2 protein levels [171]. 

Evidence exists of interactions between lncRNAs and miRNAs affecting PD-L1 expression. In prostate cancer, lncRNA KCNQ1OT1 negatively regulates miR-15a by direct binding, which in turn affects PD-L1 expression, thus promoting immune evasion [172]. In hepatocellular carcinoma, lncRNA-ATB binds to *IL11* mRNA, increasing its stability and triggering the oncogenic STAT3 signaling [173]. Competitively binding with miR-200 family members, lncRNA-ATB is also able to increase ZEB1 and ZEB2 expression levels. One of the most well-studied lncRNAs is the HOX transcript antisense RNA (HOTAIR), upregulated in several cancers including cervical cancer [174], gastric cancer [175], lung cancer [176], and breast cancer [177]. Knockdown of HOTAIR directly impairs EMT in cancer cells, reducing migration and invasion [178]. In colorectal cancer cells, HOTAIR directly binds and recruits SNAIL to regulate a transcription factor, HNF-4α [178]. However, it can also act indirectly by downregulating miRNAs, such as miR-7 a regulator of the *SETDB1* and STAT3 pathway in breast cancer [177].

## 7. Conclusions

**Figure 4:**
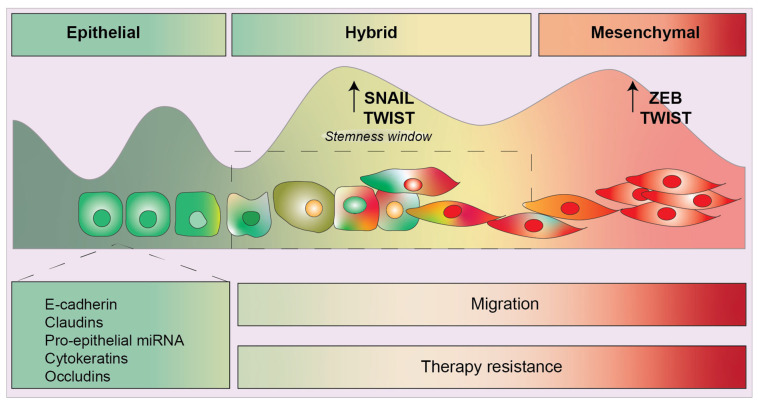
Schematic diagram displaying the several phenotypes along the epithelial to mesenchymal spectrum. Epithelial characteristics include expression of cadherins, cytokeratins, pro-epithelial miRNA, and occludins. Hybrid phenotypes give rise to a stemness/plasticity window where cells contain equal expression of epithelial and mesenchymal features. Recent studies have shown that SNAIL is important to maintain this hybrid feature whereas ZEB1 is required to fully push hybrid cells to a completely mesenchymal phenotype.

The traditional binary view of EMT has been challenged by the concept of epithelial-mesenchymal plasticity, suggesting that tumor cells can exist in intermediate hybrid E/M states (Figure 4). The expression of particular EMT-TF combinations by tumor cells determines their capacity to reside in different cell states (or hybrid E/M phenotypes). Increasingly, these intermediary states are being understood as essential for the activation of metastasis and resistance to particular agents in different cancers. Mounting research has also identified that, in addition to differential expression of EMT-TFs, the presence of particular co-factors and epigenetic regulators, miRNAs, and lncRNAs, provide additional layers of regulation to shape epithelial plasticity. Moreover, pro-inflammatory mediators such as IFNγ, IL-1β, and IL-6 also induce EMT-TFs, leading to chronic inflammation in the tumor microenvironment, immune escape, and the acquisition of EMT-like features in tumor cells. Altogether, understanding these regulatory processes provides mechanistic insights into tumor development and progression, and opens avenues for therapeutic intervention and treatment.
